# Retraction: Spiking correlation analysis of synchronous spikes evoked by acupuncture mechanical stimulus

**DOI:** 10.3389/fncom.2023.1188613

**Published:** 2023-03-28

**Authors:** Frontiers Editorial Office

Our office received a complaint claiming that the cited article is unacceptably similar to the article: Shimazaki H, Amari S-i, Brown EN, Grün S (2012) State-Space Analysis of Time-Varying Higher-Order Spike Correlation for Multiple Neural Spike Train Data. *PLoS Comput. Biol*. 8(3): e1002385. doi: 10.1371/journal.pcbi.1002385. Our investigation, conducted in accordance with Frontiers' policies, confirmed the similarity, and so the article has been retracted.

This retraction was approved by the Chief Editors of Frontiers in Computational Neuroscience and the Chief Executive Editor of Frontiers. The authors did not agree to this retraction.

